# Impacts of the COVID-19 pandemic on the health of university teachers and students: a scoping review

**DOI:** 10.3389/fpsyg.2025.1428707

**Published:** 2025-02-27

**Authors:** Magda Guimarães de Araujo Faria, Christiane Gleyce da Silva Freitas Venâncio, Fádia Carvalho Pacheco, Fabiana Ferreira Koopmans, Luciana Valadão Vasconcelos Alves, Patrícia Maia Valente

**Affiliations:** ^1^Nursing School, Rio de Janeiro State University, Rio de Janeiro, Brazil; ^2^Rio de Janeiro Municipal Health Department, Rio de Janeiro, Brazil; ^3^Center for Integrated Library Systems, National Cancer Institute, Rio de Janeiro, Brazil; ^4^Coordination of Innovation, Research and International Exchange, Fluminense Federal Institute, Rio de Janeiro, Brazil; ^5^Organ Transplant Sector, Universitätsklinikum Schleswig-Holstein, Lübeck, Germany

**Keywords:** COVID-19, universities, health promotion, mental health, health impact assessment

## Abstract

**Objective:**

To identify the impacts of the COVID-19 pandemic on the health of university professors and students. Method: Scoping review following the protocol recommended by the Joanna Briggs Institute. The research question followed the order imposed by the mnemonic “PCC” (Population, Concept, and Context), namely: what are the impacts of the COVID-19 pandemic on the health of university professors and students?

**Results:**

The analysis sample consisted of 29 texts in article format. Two main categories of health repercussions were observed, namely: category 1—repercussions on mental health; category 2—repercussions on physical health.

**Conclusion:**

It is understood that harmful effects on mental health will be an ever-present reality in the university context, since exhaustion is a characteristic of academic work itself. This situation can have serious consequences for the individual, requiring intervention projects combined with public policies that minimize these effects. It is necessary to review the role of the university in today’s society.

## Introduction

The health restrictions following the emergence of the COVID-19 pandemic gave rise to new habits and work routines across the entire population (Organização Pan-Americana de Saúde, 2022). In the university scenario, the need for social isolation virtualized teaching and research practices, requiring teachers and students to quickly adapt to the use of new technologies, without training or equipment made available by the institutions (Trust and Whalen, 2020).

Despite being indispensable, the periods of lockdown combined with emergency remote teaching revealed numerous vulnerable situations, such as the absence of electronic devices and a suitable environment for maintaining teaching activities (de Klerk and Palmer, 2021), the difficulty in handling the equipment technological mediation (Dadaczynski and Tolks, 2021), intensification of work during the period of adaptation to the new reality (Scheel et al., 2023) and even food insecurity, as many university restaurants were closed during the period (Owens et al., 2020). Such issues were also influenced by the international context of the COVID-19 pandemic, especially due to the increase in the infodemic and the maintenance of uncertainties and insecurities (Banerjee and Meena, 2021).

Adapting to the new demands arising from the period of isolation imposed by COVID-19 contributed to the increase in hours worked and to the creation of a “new normal” in which exhaustion is part of academic work (Horta et al., 2022). The COVID-19 pandemic has brought drastic changes in behavior and work processes, and the absence of similar phenomena in contemporary history makes this analysis essential for preparing society in the event of similar future events.

We understand that the global crisis caused by COVID-19 may not be the last phenomenon of this nature, and understanding the lessons learned during this period will certainly contribute to increasing resilience in future events. In addition, it is necessary to emphasize that the health repercussions evidenced in this investigation may be associated with subsequent events such as students’ intention to drop out of university (Schriek et al., 2024), increased use of psychoactive substances as a defense mechanism (Petrović et al., 2022), and even signs of suicidal ideation (Jones et al., 2023).

Therefore, the present study aims to identify the impacts of the COVID-19 pandemic on the health of university teachers and students.

## Methods

This is a scoping review, following the protocol of the Joanna Briggs Institute (Peters M. D. J. et al., 2020). The method consists of mapping studies on the topic, which aims to provide a description of the articles analyzed, not critically evaluating the evidence found, as seen in systematic reviews, but rather using identified concepts that support the research. Thus, another important characteristic is the ability to delve deeper into the topic, analyzing everything that has already been constructed, approached conceptually, to assist in future research (Peters M. et al., 2020).

The review protocol for this investigation was registered on 05/23/2023 on the Open Science Framework platform, under the link https://osf.io/3rkbg.

Using the PCC strategy (population, context and concept), the following review question was outlined: what are the impacts of the COVID-19 pandemic on the health of university teachers and students? Being the population, the students and university teachers. For a better understanding of the reader, the understanding of “university students” was established, those individuals enrolled in higher education, regardless of the course or type of institution, be it public, private or any other format. In relation to university professors, professors and researchers linked to higher education institutions were included regardless of their employment relationship (whether permanent or temporary), the course they were linked to and the type of institution.

In this concept, any and all repercussions for health were considered, be they health protective factors or illness processes of any nature, regardless of physical or mental evidence. The context of this study is the COVID-19 pandemic period from its establishment in 2020 until the end of the international health emergency established by the Organização Mundial da Saúde (2023).

### Eligibility criteria

Publications with methodological approaches comprising: qualitative, quantitative, mixed, reflections, editorials, guidelines, manuals, policies published between 2020 and 2023. The time frame occurred due to the non-existence of the COVID-19 phenomenon in time prior to the year 2020 and the end of the international health emergency established by the Organização Mundial da Saúde (2023).

### Sources and search strategies

Concept mapping was developed using controlled health vocabularies, Health Sciences Descriptors (DeCS), produced by the Latin American and Caribbean Center for Health Sciences Information/Pan American Health Organization (2022)/World Health Organization (BIREME/PAHO/WHO), and the Medical Subject Headings (MeSH), produced by the US National Library of Medicine (NLM), which can be seen in Table 1.

**Table 1:** Concept mapping.

Concept mapping		
University teachers and students	Docentes OR Faculty OR “Corps enseignant” OR Estudantes OR Students OR Estudiantes OR Étudiants	MESH/DECS Controlled Vocabulary Terms
	University Professor* OR “School Enrollment” OR “School Enrollments” OR Student*	Alternative terms in English
	“Corpo Docente” OR Docent* OR Educador* OR Professor* OR “Professor de Ensino Terciário” OR “Professor Universitário” OR “Professores de Ensino Superior” OR “Professores do Ensino Superior” OR “Professores Universitários” OR “Cuerpo Docente” OR Profesor* OR “Profesor Universitario” OR “Profesores Universitarios” OR “Corps enseignants” OR Alun* OR Estudant* OR Alumn* OR Estudiant* OR Élèves OR Étudiantes OR “Inscription à l’école” OR “Inscription scolaire”	Alternative terms in Portuguese, Spanish and French
Health repercussions	“Avaliação do Impacto na Saúde” OR “Health Impact Assessment” OR “Evaluación del Impacto en la Salud” OR “Évaluation des impacts sur la santé”	MESH/DECS Controlled Vocabulary Terms
	“Health Impact Assessments”	Alternative terms in English
	“EIS (Évaluation de l’Impact sur la Santé)” OR “Évaluation des effets sanitaires” OR “Évaluation des effets sur la santé” OR “Évaluation des impacts sanitaires” OR “Évaluation des incidences sanitaires” OR “Évaluation des incidences sur la santé”	Alternative terms in Portuguese, Spanish and French
Pandemic COVID-19	COVID-19	MESH/DECS Controlled Vocabulary Terms
	“covid-19 Pandemic” OR “covid-19 Pandemics” OR “2019 Novel Coronavirus Pandemic” OR “2019-nCoV Pandemic” OR “covid-19 Pandemic” OR “covid-19 pandemic” OR “covid-19 Pandemics” OR “Wuhan Coronavirus Pandemic”	Alternative terms in English
	“Pandemia covid-19” OR “Pandemia por covid-19” OR “Pandemias por covid-19” OR “Pandemia de covid-19” OR “Pandemia de la covid-19” OR “Pandemia del Nuevo Coronavirus 2019” OR “Pandemia por el Coronavirus de Wuhan” OR “Pandemia por el Nuevo Coronavirus (2019-nCoV)” OR “Pandemia por el Nuevo Coronavírus 2019” OR “Pandemias de covid-19” OR “Pandémie de coronavírus de 2019–2020” OR “Pandémie de covid-19” OR “Pandémies de covid-19”	Alternative terms in Portuguese, Spanish and French

With DeCS, the mapping was created using the four languages recommended by BIREME, Portuguese, English, Spanish and French. As for MeSH, the terms are exclusively in English. In both cases, the main descriptors and alternative terms were used using the Boolean operators AND (inclusion) and OR (alternative) and truncation*, which retrieves all terms with the prefix and/or suffix with the radical that accompanies it.

### Search strategy

The first stage of bibliographic search was carried out in the following databases: MEDLINE via pubmed, EMBASE, Scopus, Cumulative Index to Nursing and Allied Health Literature (CINAHL) and the Portals: PERIÓDICOS CAPES and Scielo. The second stage of the search is related to the search in gray literature that occurred in the Google Scholar database and in the Digital Library of Theses and Dissertations.

### Study selection

**Figure 1:**
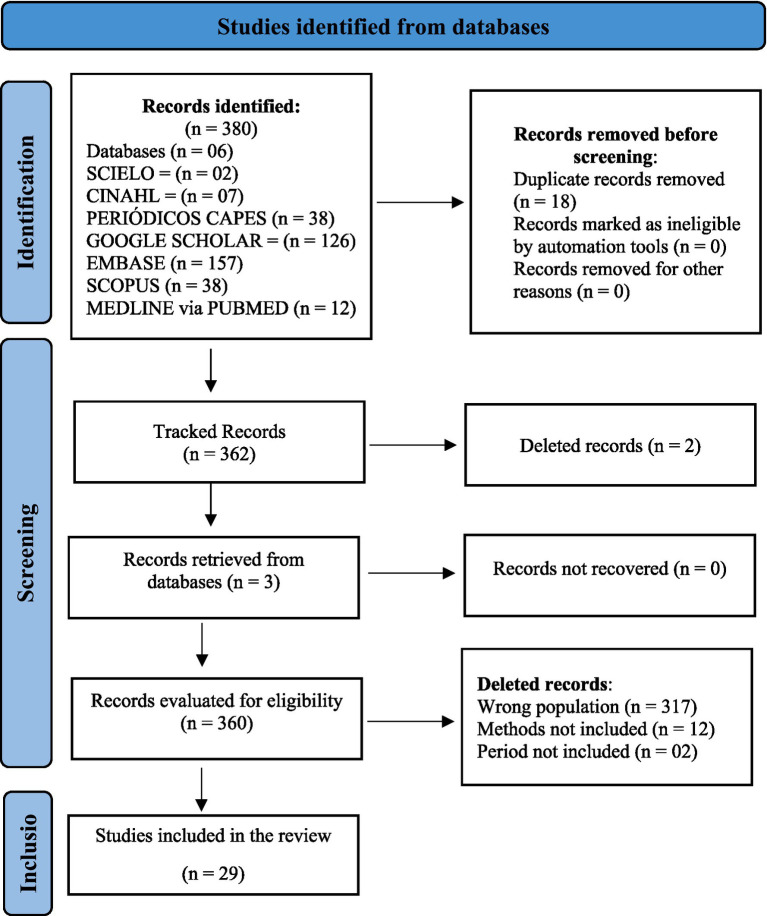
PRISMA flowchart of articles selected for inclusion in the scoping review.

The results obtained after the search were imported into the Rayyan Reviews manager (Ouzzani et al., 2016), an open access web application developed by the Qatar Computing Research Institute (QCRI). With Rayyan, the title and abstract were read blindly by three independent researchers, where those who answered the research question were chosen to read the full text. The search, exclusion and selection flow of studies can be seen in Figure 1.

### Data extraction strategies

The data were extracted and arranged in a single table composed of the following information: Identification (title, year, author, country), objective, method, population studied and repercussions of the pandemic on health.

Data extraction was performed by three independent reviewers and followed the guidelines proposed by Pollock et al. (2023) (a) Use of a standardized extraction form; (b) Conducting analysis in a pilot project to identify information that was not included in the form; (c) Group discussion with the authors to define the data to be extracted; (d) Data extraction focused solely on the review question; (e) Collective analysis of the extracted data to identify possible discrepancies.

## Results and discussion

A total of 380 records were identified in the initial search, using different sources of study references. The search for gray literature was carried out in the CAPES Theses and Dissertations Bank and on the Google Scholar platform. After the first exclusion, that is, that of duplicate texts, 362 studies were identified that were retrieved in full texts and evaluated by three independent researchers, where a final sample of 29 texts was obtained, according to the results in Table 2.

**Table 2:** Characterization of selected studies.

Objective	Population and sample size	Method	Health repercussions
Identify and compare the impact of COVID-19 on the exam of final year medical students from two universities with scores prior to the pandemic period (Aaraj et al., 2022)	118 final year students from Shifa Tameer and Millat Universities were evaluated.	Cross-sectional study using validated analysis instruments.	Perception of stress and anxiety.
Assess the impact of COVID-19 on lifestyle in a sample of Pakistani medical students (Haider et al., 2020)	Online study carried out with 1,100 Pakistani medical students.	Cross-sectional online study using a self-administered questionnaire	Modifications in lifestyle, ranging from nutritional to social behavior impairment.
Understanding the impact of vaccination on stress levels among medical students (Huda et al., 2022)	58 medical interns participated in the study.	Cross-sectional study carried out in the hospital setting, using validated scales.	Increase in the stress level of interns.
Investigating the associations between stress response and mental health status among college students during the early phase of COVID-19 in China (Xu and Huang, 2022)	1,351 university students from China.	Online cross-sectional survey. It was based on a quantitative proposal using descriptive statistical analysis.	Students’ negative emotions point to an apparent relationship with mental health and cognition.
IIdentify the repercussions of the first COVID-19 lockdown on the lives of higher education students, teachers and staff (Nowrouzi-Kia et al., 2022)	The survey included 27,804 participants from 121 countries and 6 continents, including higher education students, teachers and staff around the world.	Global online cross-sectional questionnaire survey using a mixed-method design and disseminated to college students, faculty, and staff from April to November 2020.	Increased stress and anxiety levels and decreased quality of life during the first 2 weeks of lockdown.
Identify academic burnout and problematic smartphone use during the COVID-19 pandemic (Hao et al., 2021)	748 Chinese undergraduate students participated in the study.	Cross-sectional quantitative research. Data collection was carried out online using validated scales and subsequent statistical analysis.	Academic burnout is related to the compulsive use of smartphones, especially due to anxiety.
Investigate the effect of COVID-19 on student mental health and related factors (Hekmat et al., 2021)	Eight articles were selected and evaluated quantitatively and qualitatively.	Systematic literature review	Increased levels of stress, anxiety and depression.
Explore the impact of COVID-19 on the mental health of optometry students at a higher education institution (Simjee et al., 2021)	147 students currently enrolled at a South African university.	Cross-sectional study using the validated DASS-21 scale. Interview data were analyzed using thematic content analysis.	Increased levels of stress, anxiety and depression.
Identify the relationship between stress factors, general health and academic performance (Al-Rouq et al., 2022)	421 medical students participated.	Online survey using a validated perceived stress scale.	Elevated stress levels in their final educational years.
Investigate the impact of the COVID-19 pandemic on the mental health and sleep of university students in Saudi Arabia (Alyoubi et al., 2021)	582 university students in Saudi Arabia participated.	Online and cross-sectional questionnaire assessing depression, anxiety, stress, resilience and insomnia during the COVID-19 pandemic.	Indication of high levels of depression, anxiety and stress and low levels of resilience.
Assess the mental situation of college students during the epidemic (Cao et al., 2020)	7,143 university students participated.	Cross-sectional investigation carried out using anonymous structured questionnaires.	Noticeable impairment in the mental health of university students.
Assess the impact of COVID-19 on medical student internships in public and private institutions in Brazil (Carrascosa et al., 2020)	317 Brazilian medical students participated.	Cross-sectional quantitative research with online data collection and subsequent statistical analysis.	The presence of feelings and symptoms of anxiety was observed.
Analyze the impact of the COVID-19 lockdown on BMI changes. Sleep quality and time spent on physical activities in an Italian academic community of students and workers (Cremasco et al., 2021)	Academic community in Northern Italy, with 2,838 students and 828 workers.	Cross-sectional online survey was distributed in May 2020, after 2 months of lockdown in Italy.	An increase in BMI, worsening of nighttime sleep quality and daily excess of sedentary activities were observed.
Investigate university students’ perceptions of their learning process and the influence on mental health during the COVID-19 pandemic period (El-Sakran et al., 2022)	A total of 588 university students in the UAE.	Cross-sectional quantitative research with online data collection, use of validated scales and subsequent statistical analysis.	The occurrence of psychological suffering, anxiety, anguish and financial problems caused by COVID-19 is observed.
To analyze the educational, emotional and social impact of the state of emergency period imposed by the COVID-19 pandemic on Romanian university students (Gavriluță et al., 2022)	1,013 Romanian university students participated.	Cross-sectional quantitative research with online data collection, use of validated scales and subsequent statistical analysis.	Feelings of loneliness, panic, fear and aggression were observed.
Identify the impact of COVID-19 on the assessment exams of medical students (Harky et al., 2020)	46 medical students from a UK university participated.	A retrospective observational study, using a questionnaire.	The impacts were diverse, with an emphasis on students’ mental health.
Assess factors associated with increased levels of mental health burden among North American college students during COVID-19 (Kecojevic et al., 2020)	162 college students in northern New Jersey, the US region, participated.	Cross-sectional research that collected with multivariate regression analysis.	High levels of mental suffering were observed, with an emphasis on depression and anxiety.
Investigate the mental and psychological impact of the COVID-19 pandemic and associated factors on a sample of Portuguese higher education students (Laranjeira et al., 2022)	1,522 Portuguese higher education students participated.	Online cross-sectional study with convenience sampling.	Moderate prevalence of feelings of hopelessness and lack of resilient coping.
Describe the use of telepsychiatry and the acceptance of medical students to use it for their mental health needs (Lavergne and Kennedy, 2021)	Not applicable		The prevalence of anxiety and stress symptoms was observed.
Identify the psychological impact of COVID-19 on university students in Wuhan (Li et al., 2021)	4,355 university students from Wuhan - China participated.	Online cross-sectional quantitative research carried out in April 2020.	High severity emotional and mental impacts were observed.
Identify the repercussions of COVID-19 for college students at a minority-serving academic institution in New York (López-Castro et al., 2021)	909 college students from New York City participated.	Cross-sectional quantitative research with online data collection, use of validated scales and subsequent statistical analysis.	The presence of anxiety and stress disorders was observed.
Understanding the impact of COVID-19 on teachers at the Faculty of Medicine of the University of Porto-Portugal (Miguel et al., 2021)	Medicine professors at the University of Porto-Portugal	Cross-sectional, quantitative, qualitative and analytical online study using validated scales.	High levels of exhaustion were observed.
Assess the impact of isolation on the health and comfort of college students and the role that home characteristics may have played (Millán-Jiménez et al., 2021)	188 medical and architecture students from a Spanish University participated.	Observational, descriptive and cross-sectional study.	The presence of anxiety symptoms and increased alcohol consumption was observed.
Analyze the psychological impact of COVID-19 on the university community during the first weeks of confinement (Odriozola-González et al., 2020)	2,530 members from a Spanish university participated.	Cross-sectional investigation carried out with an online questionnaire sent in March 2020.	There was an intense impact on the mental health of students and workers.
Evaluate the impact of the three blocks on the mental health and engagement of doctoral students during the construction of the thesis and evaluate the protective role of self-compassion in depression, anxiety and stress (Paucsik et al., 2022)	134 PhD students from three French universities participated.	Longitudinal research using online questionnaires.	An increase in depression, anxiety and stress was observed, as well as a significant decrease in wellbeing and engagement with the doctorate during the first year of the pandemic.
Explore student perceptions of COVID-19 risks and preventive measures by assessing the health, social and financial impacts of national lockdown measures on students in Uganda (Rawlings et al., 2022)	398 undergraduate and postgraduate students in Uganda participated.	Cross-sectional quantitative research with online data collection, use of validated scales and subsequent statistical analysis.	Students indicated their perception of depression, frustration, stress and anxiety during the lockdown; they became less physically active and spent most of their time on social media.
Assess changes in the mental health and wellbeing of medical and nursing students 1 year after COVID-19 (Richardson et al., 2022)	2,275 students studying medicine or nursing at UK universities participated.	Cross-sectional and multi-institutional study using an online questionnaire.	The existence of anxiety disorders, stress and fear was noted.
To evaluate the impact of headache symptoms in a population of Italian university students during the period COVID-19 (Saracco et al., 2022)	4,926 university students from all over Italy participated.	Cross-sectional study using an online questionnaire.	The negative impact of stress was observed, with repercussions on physical symptoms such as headache.
Investigate perceptions and expectations about the health of university students’ physical living environments and the implications for health (Zhao et al., 2022)	301 university students studying in Zhejiang, China participated.	Cross-sectional study using an online questionnaire.	The presence of physical and mental repercussions was observed.

There was a preponderance of studies conducted in countries of the global north, while studies conducted in developing countries were scarce. This scenario is believed to be due to limitations in research funding. Furthermore, there was a lack of qualitative research, which can be explained by the period of social distancing, which made the use of online forms the most viable way to obtain research data.

**Figure 2:**
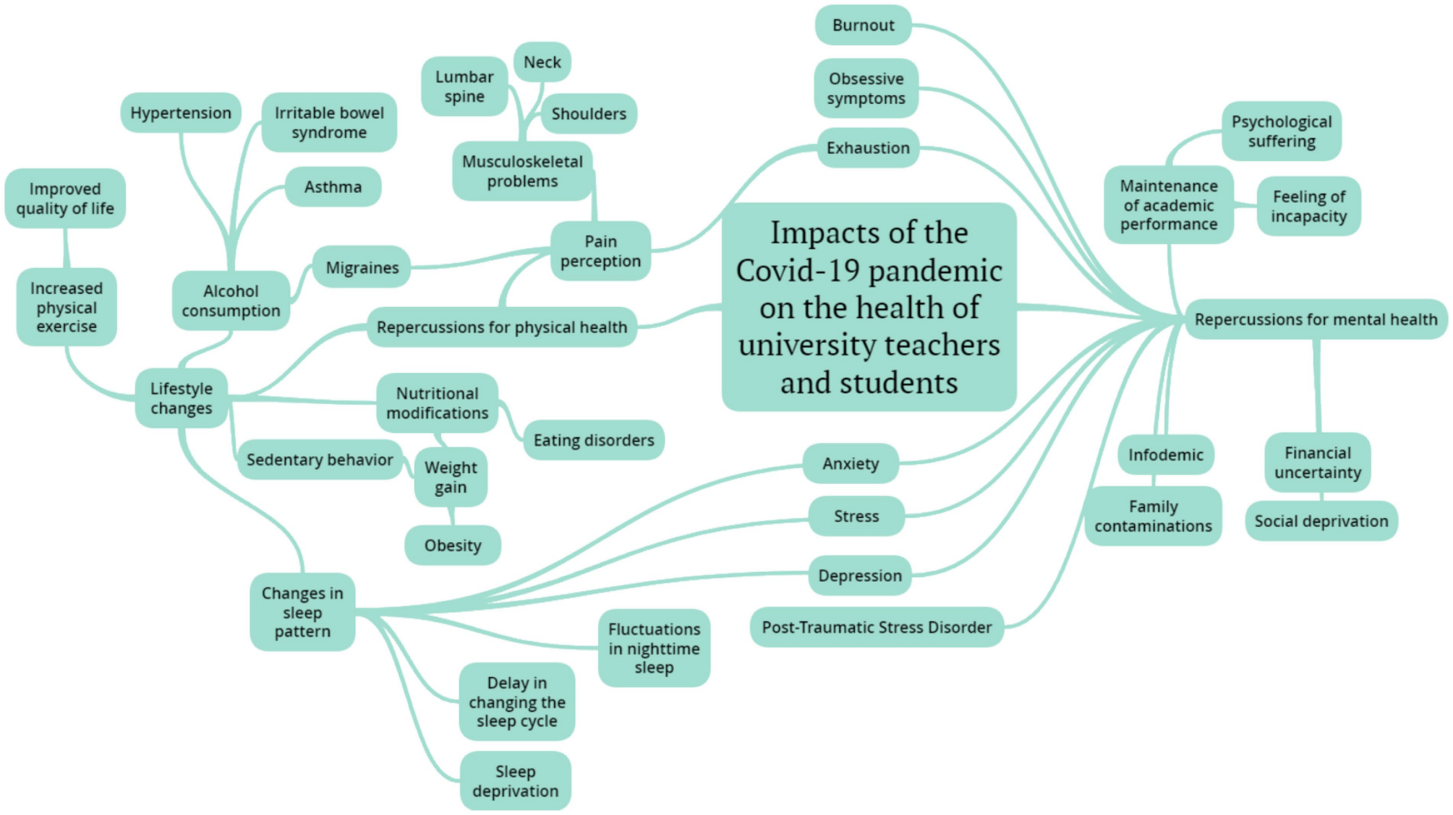
Synthesis of the relationships between physical and mental repercussions of COVID-19 on the health of the university population.

Data analysis helped identify themes with greater relevance to the study, namely: (1) Repercussions for mental health and; (2) Repercussions for physical health. The synthesis of the relationship between these themes can be seen in Figure 2 and, later, a discussion of the findings will be presented.

### Repercussions for mental health

It is observed that most of the findings refer to repercussions on mental health, whether by worsening a pre-existing mental impairment or as the emergence of a new comorbidity, due to the pandemic period (Aaraj et al., 2022; Al-Rouq et al., 2022; Alyoubi et al., 2021; Cao et al., 2020; Carrascosa et al., 2020; El-Sakran et al., 2022; Gavriluță et al., 2022; Hao et al., 2021; Harky et al., 2020; Hekmat et al., 2021; Huda et al., 2022; Kecojevic et al., 2020; Laranjeira et al., 2022; Lavergne and Kennedy, 2021; Li et al., 2021; López-Castro et al., 2021; Miguel et al., 2021; Millán-Jiménez et al., 2021; Nowrouzi-Kia et al., 2022; Odriozola-González et al., 2020; Paucsik et al., 2022; Rawlings et al., 2022; Richardson et al., 2022; Saracco et al., 2022; Simjee et al., 2021; Xu and Huang, 2022; Zhao et al., 2022).

The pandemic and confinement had a major impact on educational institutions around the world and perhaps this was the first major phenomenon on a global scale to affect educational processes, given the need to quickly adapt to emergency remote teaching (Nowrouzi-Kia et al., 2022). Furthermore, in the student context, the uncertainties of the future were compounded by the maintenance of academic performance and the identification of neglected content in the virtual context, which required effort in the search for complementing knowledge, contributing to the psychological suffering of this population (El-Sakran et al., 2022; Harky et al., 2020).

In this sense, the change in the learning process had an impact on students’ confidence in their own training, leading to a feeling of incapacity. Only 54% of health students felt able to take on care activities for other human beings (Carrascosa et al., 2020). Another important point was the loss of student identity with their own training and with the institution of attachment (Gavriluță et al., 2022).

Among the most frequent mental health problems among university students, stress, anxiety and depressive symptoms stand out (Araújo et al., 2020). The first two may have common characteristics such as a complex combination of emotions, such as: fear, apprehension and worry, which may arise as a brain disorder or associated with other pre-existing problems (Asif et al., 2020). Depressive symptoms can worsen with an increase in depressed mood, decreased energy and limited interest in day-to-day activities (Haider et al., 2020). It is worth noting that pre-existing mental health conditions may have contributed to the increase in the aforementioned symptoms among the general population (Shigemura et al., 2020) and among university students (Alyoubi et al., 2021; Kecojevic et al., 2020). Dissatisfaction with the learning environment was the main reason for these symptoms (Harky et al., 2020; Paucsik et al., 2022).

In an initial attack, a significant correlation was observed between negative emotions caused by the pandemic and the perception of stress symptoms (Xu and Huang, 2022), impacting academic performance, which in turn, increased perceived stress (Aaraj et al., 2022).

In relation to anxiety disorders, it was observed that the mass movement of information contributed to the emergence or worsening of the condition in the population (Sher, 2020). Among higher education students, symptoms of anxiety and stress appeared at a higher incidence than in the rest of the population and were aggravated by the temporal barriers to seeking specialized help (Lavergne and Kennedy, 2021). This situation is even more serious when related to students in the health area, as they face a greater risk of exposure and increased workload, in addition to setbacks and thinking dilemmas during clinical teaching, leading to an increased risk of psychological disorders (Huda et al., 2022).

The time of exposure in a risk situation is also a rival in the quality of life of students and, in this sense, a progressive increase in stress, anxiety and depression scores was observed throughout the confinements caused by the COVID-19COVID-19 pandemic (Essadek and Rabeyron, 2020). Academic life is permeated with the sociability inherent to the university context and the deprivation of this experience, combined with the demands of the moment, contributed to the development of specific symptoms (Juniper et al., 2012). Furthermore, as mental health care was delayed and rescheduled, individuals became committed to dealing with the long-lasting repercussions of the pandemic (Richardson et al., 2022).

The prolongation of the pandemic also increased the risk of having your support network infected by COVID-19, which proved to be an independent risk factor for anxiety, as well as financial uncertainty (Cao et al., 2020).

It was possible to observe significantly higher levels of Post-Traumatic Stress Disorder (PTSD) in university students compared to people with other occupations (Li et al., 2021). The high prevalence of PTSD may reflect multiple and cumulative stressors that students face at individual, relational and more distant social levels (López-Castro et al., 2021). Fear also intensified obsessive symptoms, where it was observed that individuals diagnosed with Obsessive Compulsive Disorder (OCD) experienced an increase in the frequency and intensity of concerns about contamination and cleanliness related to COVID-19 (Haider et al., 2020).

Contrary to literature findings, some studies have identified normal levels of depression, anxiety and stress among students during the period of isolation and the causes for this phenomenon may be related to the cultural and social aspects of this specific group (Laranjeira et al., 2022; Simjee et al., 2021).

In line with the student context, the repercussions for teachers’ mental health contributed to a feeling of exhaustion, especially related to emergency remote teaching (Mariño-Narvaez et al., 2021). A burned-out faculty member may experience anxiety, irritability, and sadness, which can lead to somatic symptoms such as sleep disturbances, headaches, digestive problems, and alcohol and drug abuse. There is a combination of factors that make burnout a complex and multifactorial phenomenon (Ramos et al., 2023).

Adaptations to remote work required the teacher to adapt to a new work dynamic, which often exceeded the negotiated workload, whether in synchronous classes or asynchronous activities and continuous availability on messaging applications (Saraiva et al., 2020). The abrupt change of their teaching activities to the remote environment, at the same time that they experienced insecurity, fears and uncertainties related to the possibility of contagion and death related to the pandemic, further increased the frequency of negative feelings and mental illnesses (Baptista et al., 2023).

In this sense, around 41.2% of teachers suffered serious personal burnout and 16% of teachers suffered serious student-related burnout. These teachers tend to be less involved in teaching activities, thus impacting the learning process. Furthermore, life satisfaction and resilience appeared to be a protective variable, while stress and changes in sleeping habits were negatively associated with burnout among teachers (Miguel et al., 2021).

The negative impacts are not only on mental health, but also on social life and the quality of life of the social body in higher education around the world, hindering productivity processes (Nowrouzi-Kia et al., 2022). Social isolation intensified the feeling of frustration, increasing social vulnerabilities and catalyzing the processes of psychological suffering (Lima et al., 2022; Rawlings et al., 2022).

The development of mental health protection factors, such as strengthening social support for the academic group and promoting coping mechanisms in crisis situations, help the university population in difficult times (Paucsik et al., 2022). In this sense, university and health system management seem to be preponderant for adequate psychological counseling that can, in the long term, reduce the negative impact of COVID-19 on mental health (Hekmat et al., 2021). The construction of new service protocols, online psychological interventions or psychological interventions aimed at specific groups are considered necessary measures (Odriozola-González et al., 2020).

### Repercussions for physical health

Regarding the physical health of the university social body, evidence was also found of the negative impacts resulting from the COVID-19 pandemic, resulting in direct changes in lifestyle and pain perception (Cremasco et al., 2021; Haider et al., 2020; Millán-Jiménez et al., 2021; Zhao et al., 2022).

The changes that occurred during this period were very significant in the lifestyle of the entire academic community. Weight gain, for example, was present in 38% of a certain sample of Spanish students (Millán-Jiménez et al., 2021). The results were similar to a general Brazilian sample, where an increase in body mass index (BMI) was noticed in almost 20% of respondents (Costa et al., 2021). Obesity can be understood as a direct risk to health, whether in the emergence of chronic conditions or in the congestion of the health service in the face of the assistance actions necessary to maintain the quality of life of this population (Bolsoni-Lopes et al., 2021).

The constant bombardment of news and information in the media about the pandemic can be stressful and consequently lead people to overeat, especially when looking for foods that help relieve stress (Mattioli et al., 2020), however, the practice of Diets were common during the isolation period and were associated with the risk of eating disorders (Andrade et al., 2021).

Weight gain during the pandemic is associated with an increase in the quantity and quality of food eaten (Balanzá-Martínez et al., 2020). It is interesting to note that the main leisure activity of the university population was associated with sedentary behavior, such as passive entertainment in front of screens. In this sense, physical activity was restricted to between one and 6 h per week, contributing to weight gain associated with low physical activity and to an increased risk of developing physical and mental problems (Cremasco et al., 2021; Ferrara et al., 2022).

On the other hand, performing physical activities was associated with a protective factor against psychological symptoms, whether among students or university employees (Nowrouzi-Kia et al., 2022). Furthermore, it is noteworthy that individuals who already maintained an exercise routine before the pandemic and maintained this commitment, had their quality of life less affected, while those who exercised and stopped practicing during the pandemic period, had worse rates even when compared to previously sedentary people (López-Castro et al., 2021).

Moments of rest also had a negative impact on the overall health of the studied population, as poor sleep quality was often associated with greater quantity and worse quality of sleep, with patterns such as: fluctuations in nighttime sleep, delay in changing the sleep cycle, sleep and sleep deprivation, being more prevalent among individuals who suffer from anxiety, depression or stress (Cremasco et al., 2021; López-Castro et al., 2021).

Another phenomenon with physical repercussions was alcohol consumption during the COVID-19 pandemic, reaching 59% of consumption (Millán-Jiménez et al., 2021). Despite being considered a high percentage, the increase in alcohol intake by university students was 11.79% (Oliveira et al., 2022). In addition to the slight increase in alcohol consumption, it was observed that students with reports of comorbidities had reported conditions such as hypertension, asthma, irritable bowel syndrome, headache and migraines (Al-Rouq et al., 2022).

The negative effect of regular headaches on academic performance was confirmed by general performance in academic work and only 20% of affected students sought specific treatment (Saracco et al., 2022).

Added to this, it is understood that the carrying out of work activities in the domestic environment often came up against the lack of structure for their development, the increase in overload related to the extension of the working day and the lack of rest and leisure for teachers and university students, which harmed mental health as well as physical health for ergonomic reasons (Geller et al., 2023; Huang and Zhao, 2020; Rodrigues et al., 2020).

Musculoskeletal problems have become common and adapting to the pandemic scenario has resulted in great difficulties and limitations, leading to physical and emotional exhaustion on the entire academic staff. The body regions with the highest prevalence of pain complaints were the lumbar spine (60%), neck (56%) and shoulders (48%) (Kraemer et al., 2020; Šagát et al., 2020).

It is worth highlighting that low back pain could be directly related to inadequate posture when carrying out work activities (Kraemer et al., 2020; Matias et al., 2022), while pain in the neck, shoulders, arms and wrists was related to prolonged exposure to the use of electronic devices (Gammoh, 2021).

## Limitations of the study

The data collection for this study was carried out immediately after the end of the COVID-19 health emergency and we understand that the number of texts on the subject tends to grow, especially because we are still experiencing the repercussions of COVID-19. Therefore, we understand that this subject is inexhaustible, making it impossible to capture all existing bibliography.

## Conclusion

The study addresses the impacts of the pandemic on the physical and mental health of university teachers and students, highlighting the rapid transition to remote teaching and its consequences. Psychological impairments, including anxiety, stress and sleep disorders, as well as physical symptoms such as sedentary lifestyle and pain, are highlighted.

The need for interventions and public policies to mitigate the effects of the pandemic in the medium and long term is highlighted, and it is essential to reassess the role of universities in promoting health. Although there are challenges, initiatives such as the international networks of healthy universities and other similar groups indicate paths for sustainable actions.

This research serves as a basis for understanding the negative repercussions of the pandemic on the health of the university community, but new research is suggested to determine viable strategies for health recovery, focusing on the local, epidemiological, social and political characteristics of the affected population, especially in developing countries as these have presented a low number of research on the subject.
